# Normative values for the bath ankylosing spondylitis functional index in the general population compared with ankylosing spondylitis patients in Morocco

**DOI:** 10.1186/1471-2474-13-40

**Published:** 2012-03-21

**Authors:** Ghizlane Wariaghli, Fadoua Allali, Kenza Berrada, Zineb Idrissi, Ihsane Hmamouchi, Redouane Abouqal, Najia Hajjaj-Hassouni

**Affiliations:** 1 Rheumatology Department, El Ayachi Hospital, Salé, Ibn Sina University Hospital, Rabat, Morocco; 2 Laboratory of Biostatistics, Clinical Research and Epidemiology, Faculty of Medicine and Pharmacy, Rabat, Morocco

## Abstract

**Background:**

The Bath Ankylosing Spondylitis Functional Index (BASFI) has been commonly used in rheumatology to quantify functional disability in patients with Ankylosing Spondylitis (AS). Our aim was to evaluate the discriminating power of BASFI and determine the best cutoff score of this index in the general population compared with AS patients.

**Methods:**

A cross-sectional study that included 200 patients suffering from AS and 223 subjects from the general population matched for age and sex was carried-out. The discriminating power of the BASFI by strata of age was evaluated by the area under the Receiver Operating Characteristic curve and the best cutoff was determined by the Youden index.

**Results:**

The mean age of the general population was 39 ± 12 years. 76.7% of them were male. The median BASFI of the healthy subjects and patients was 0.2 and 4.5 (P < 0.001) respectively. The best cutoff of BASFI was 1.5 with a sensitivity of 86% and a specificity of 90%. In the age group of 18-29 years, the best cutoff of the BASFI was 0.9 with a sensitivity of 93% and a specificity of 94%. In the age group of 30-50 years, the best cutoff of the BASFI was 1.5 with a sensitivity of 84% and a specificity of 88%. For those over 50 years of age, the best cutoff of the BASFI was 2.5 with a sensitivity of 84% and a specificity of 97%.

**Conclusions:**

This study suggests that the discriminating power of BASFI is considered good at any age. The best cutoff of this index increased as age increases as functional disability is associated in part with lifestyle choices and increases with age. The cutoff values of the BASFI that we have presented could be used as a reference benchmark for both clinical practice and research.

## Background

The Bath Ankylosing Spondylitis Functional Index (BASFI) was developed more than a decade ago to measure functional disability of patients suffering from Ankylosing Spondyltis (AS) [1] and subsequent outcomes following therapy. The clinimetric validity and the usefulness of BASFI in cohort studies of patients with spondyloarthopathies are firmly established [2-4]. This index can contribute markedly to efficacy evaluations in cohort studies, as it exhibits accurate sensitivity to change and has been shown to have an acceptable test-retest reliability and internal validity [5]. Previous studies have shown the validity of several translations of BASFI, including the Arabic and Moroccan dialectal version [6,7] for use in our patients with AS. The Assessment of SpondyloArthritis international Society (ASAS) recommends BASFI [1] or Dougados Functional Index (DFI) [8] for the assessment of physical functioning in patients with AS [9,10]. The clinimetric properties of these instruments have been shown to be acceptable [1,4,11]. Disability according to BASFI has been studied in a large population of AS subjects [4,12,13]. However, disability measured by BASFI has not been reported in the general population of adults. Therefore, we conducted a cross-sectional study to evaluate the discriminating power of BASFI and determine the best cutoff score of this index in the general population compared with AS patients living in the same area and matched for age and sex.

## Patients and methods

### Patients with AS

El Ayachi Hospital is the main Moroccan rheumatology university center in the Rabat -Sale area. All AS patients refereed to this center for diagnosis or follow-up during January to April 2010 were recruited for this study. Patients with AS fulfilled the modified New York criteria [14]. Patients under 18 years of age were excluded from the study.

### Controls

300 healthy subjects from the general population were investigated. In order to match the AS patient group by age and sex, the general population sample was designed to have a mean age of 39 years and to include 75% male, but was otherwise random. All subjects in the control group were living in the same geographic area and were recruited by announcements at the Faculties of the University of Rabat, hospitals, in various public institutions and senior residences. Healthy subjects diagnosed with any pulmonary, cardiovascular, neurological or rheumatological comorbidity affecting their ability to do daily activities were excluded from the study. Subjects who replied to the announcements were selected so that 223 subjects from the general population were analyzed within each of the following age categories: 18-29, 30-50, and > 50 years. Subjects in the control group were matched for age and sex to AS patients. Within each age-sex stratum, the sampling process was purely random assuring representativeness.

The study protocol was approved by the ethics committee of the Mohamed V University in Rabat (Faculty of Medicine and Pharmacy). All patients and healthy controls gave written informed consent before participating in the study according to the Declaration of Helsinki.

The subjects completed a questionnaire comprising a complete copy of BASFI and information on socio-demographic characteristics such as age, sex, height and weight for calculating the body mass index (BMI; weight (kg)/height (m^2^)), smoking status, level of education and physical activity. Patients with AS were also asked to fill out a questionnaire consisting of the Bath Ankylosing Spondylitis Disease Activity Index (BASDAI) score (0-10), nocturnal and global pain assessed on a 0-100 mm visual analog scale (VAS) and disease duration.

### Bath ankylosing spondylitis functional index

BASFI is a visual analog scale (VAS) index, whereby the patient rates his/her ability to perform tasks (BASFI) by marking a vertical line on a 100 mm horizontal line. It consists of ten tasks lines to assess the degree of difficulty of performing each task. The tasks are: (1) putting on socks, (2) bending forward to pick up a pen, (3) reaching a high shelf, (4) getting up from an armless chair, (5) getting up from the floor from lying supine, (6) standing unsupported, (7) climbing steps without a handrail, (8) looking over their shoulders, (9) performing physically demanding activities, and (10) doing a full day's activities. The total BASFI score is calculated by adding all ten scores and dividing by 10. The validity and reliability of the Moroccan version of BASFI has been established previously [6].

### Statistical analysis

Descriptive statistics were used to describe the study sample. Differences in mean and median BASFI between the two groups (patients and general population) were determined using Student's *t*-test and Chi Square test. The discriminating power of BASFI by strata of age was evaluated by the area under the Receiver Operating Characteristic (ROC) curve [AUC] and the best cutoff point was determined by the Youden index (better sensitivity and specificity). Analyses were performed with SPSS Software version 10,0

## Results

### Characteristics of patient population

A total of 200 patients with AS were recruited for this study. The mean age of the patients was 39.1 years (Standard deviation (SD) 12.6; range: 18-67 years). 76.5% of the selected subjects were male, and the mean disease duration was 10.6 years (SD 7.9). At the time of the study, the level of global and nocturnal pain evaluated on a 0-100 mm VAS was 46 (SD 23) and 39 (SD 29) respectively and the mean BASDAI was 4.08 (SD 2.23). A total of 90% of the patients were on non-steroidal anti-inflammatory drugs while 15% of the patients were being treated by anti-tumor-necrosis factorα.

### Characteristics of control population

A total of 223 control subjects were enrolled in the study. The mean age of the controls was 39 years (SD 12 years). 76.7% of them were male. The characteristics of patients and controls are shown in Table 1.

**Table 1:** Characteristics of AS patients and controls

	AS Patients	Controls	*P*
	(n = 200)	(n = 223)	
Age, mean ± SD years	39.1 ± 12.6	39 ± 12	NS
Age groups (years), No			
18-29	58	69	NS
30-50	103	106	NS
> 50	39	48	NS
Male sex, n (%)	153 (76.5)	171 (76.7)	NS
BMI, mean ± SD years	28.2 ± 5.7	29.7 ± 5.3	0.01
Disease duration, mean ± SD years	10.6 ± 7.9	-	
Global pain, (VAS, 0-100 mm)	46 ± 23	-	
Nocturnal pain, (VAS, 0-100 mm)	39 ± 29	-	
BASDAI (0-10 scale), mean ± SD	4.08 ± 2.23	-	
BASFI (0-10 scale), median (range)	4.5 (1-10)	0.2 (0-4.7)	< 0.001

AS: ankylosing spondylitis, BASDAI: Bath Ankylosing Spondylitis Disease Activity Index, BASFI: Bath Ankylosing Spondylitis Functional Index, BMI: body mass index, NS: not significant, VAS: visual analog scale.

### Comparison of disability

Overall, the median BASFI of the healthy subjects and patients was 0.2 (range 0-4.7) and 4.5 (range 1-10) (P < 0.001) respectively. In the control group, the median BASFI for those between 18 and 29 years of age was 0.0 (range: 0-2). It increased to 0.2 (range: 0-4.7) in the age-group of 30-50 years and to 0.3 (range: 0-3.9) in controls aged more than 50 years.

**Figure 1:**
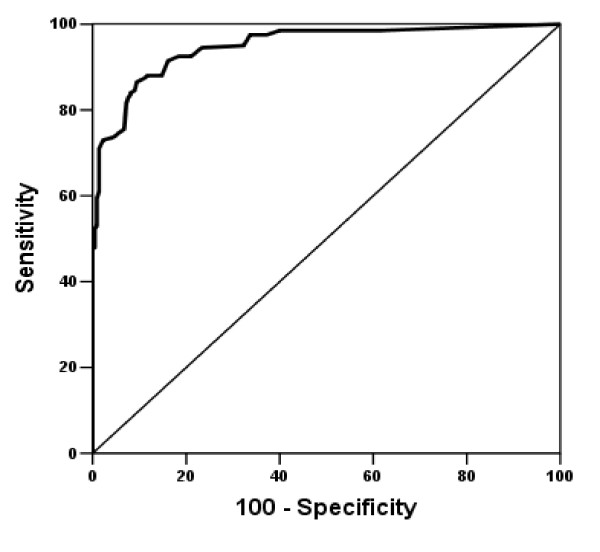
**ROC curve illustrating the discriminating power of the BASFI between AS patients and general population**.

Overall, the area under the ROC curve (AUC) (95% confidence interval [CI]) of BASFI was 0.94 (0.92-0.96). The best cutoff was 1.5 with a sensitivity of 86% and a specificity of 90% (Figure 1).

The discriminating power and the cutoff points of the BASFI in the general population compared to AS patients according to the ROC curve were evaluated in age stratum (Table 2)

**Table 2:** The discriminating power of the BASFI by age stratum according to the ROC curve

	AUC (CI:95%)	Cutoff point	Sensitivity (%)	Specificity (%)
BASFI; 18-29 years	0.96 (0.93 - 0.99)	0.9	93	94
30-50 years	0.92 (0.89 - 0.96)	1.5	84	88
> 50 years	0.96 (0.93 - 0.99)	2.5	84	97

AUC: area under the curve ROC, BASFI: Bath Ankylosing Spondylitis Functional Index; CI: confidence interval, ROC: Receiver Operating Characteristic

### Relationship between BASFI, pain, disease activity (BASDAI), BMI, education, and physical activity

In the patient group, BASFI was significantly correlated with pain score (r = 0.47; *P *< 0.001), the BASDAI (r = 0.58; *P *< 0.001), physical activity (r = - 0.21; P = 0.02) and with the BMI (r = 0.22; *P *= 0.02). However, no significant correlation between BASFI, age, sex, smoking status and level of education was noted. In the general population, functional disability (BASFI) was highly correlated with female sex (r = 0.20; *P *= 0.002), increasing age (r = 0.24; *P *< 0.001) and education (r = - 0.31; *P *< 0.001). A significant correlation was observed between BMI and BASFI (r = 0.16; *P *= 0.01) in both men and women.

## Discussion

Although BASFI is a widely applied tool to measure functional impairment in patients with AS, the comparability of BASFI across clinical populations has been hampered by the absence of normative data. Moreover, differentiation between age-related declines in functioning and those that are disease-related requires normative data (i.e., BASFI in 'normal' general populations as opposed to patient groups). So, we concluded that it made sense to identify levels of disability that are higher than could reasonably be expected in the general population. This will help the clinician to differentiate between the disability resulting from the disease versus the "normal-for-age" decrease in functioning. For example, if a man with AS aged 35 years old and a 75 year -old man with the same disease had a BASFI score of 3, would that mean that the level of disability due to their AS was the same? Indeed, both had some difficulties in their day-to-day activities but it was not possible to determine whether the level of the disability in these cases was different from that of people of the same age and sex in general. Therefore, the reference values of BASFI in the general population presented in this study may contribute to better understanding of BASFI scores in patients with AS. At present, the capacity to benchmark a patient's health status against that of age- and gender matched peers in the general population exists only for the SF-36 [15], HAQ [16,17], WOMAC, and AUSCAN indices [18]. Thus, our report is among the few in the English medical literature that identifies the discriminating power of BASFI after stratification by age and determines the best cutoff of this index in the general population compared with AS patients. Results were expressed using a ROC curve and cutoffs for designating a significant level of disability.

Overall, the best cutoff of BASFI was 1.5 with a good sensitivity and specificity. This result indicates that disability according to BASFI (0-10) is defined as a BASFI of more than 1.5 irrespective of age and sex. If we divide the population based on age-groups, we observed age-related increases in disability, which is congruent with previously published observations with the SF-36, HAQ, WOMAC, and AUSCAN indices which show similar trends, particularly when the age increases. In fact, in the age group of 18-29 years, the best cutoff of BASFI permitting to discriminate patients and subjects from the general population was 0.9 with an acceptable sensitivity and specificity. This cutoff of disability increases to 1.5 in the age group of 30-50 years and to 2.5 in the age group > 50 years.

There is a little consensus on what constitutes the cutoff score for designating a significant level of disability. We believe that any of the mean values as well as the median and other percentile values of the general population BASFI can be used as benchmarks to define disease-attributable disability in clinical populations. However, until now, BASFI scores of the general adult population have not been reported, nor have BASFI scores of patients with AS been compared with those of the general population.

## Conclusion

This study suggests that the discriminating power of BASFI is considered good at any age. The best cutoff of this index increased as age increases as functional disability is associated in part with lifestyle choices and increases with age. The normative values of BASFI that we have presented could be used as a reference benchmark for clinical studies using this index of disability.

The main limitation lies in the procedures used to select subjects, who were all volunteers and ambulatory. Another limitation of this study was the small number of healthy subjects from the general population. Therefore, we were not able to give reference values of BASFI score for all adult age groups. Then, reference values of BASFI should be evaluated in other populations and countries to confirm our results.

## Competing interests

The authors declare that they have no competing interests.

## Authors' contributions

FA and WG conceived the study and supervised its design. KB, ZI, IH participated in the data collection. RA did data management and statistical analyses. WG wrote the paper with input from FA. NHH participated in the design of the study and its execution. All authors read and approved the final manuscript.

## Pre-publication history

The pre-publication history for this paper can be accessed here:

http://www.biomedcentral.com/1471-2474/13/40/prepub
